# Regulation of chromatin states and gene expression during HSN neuronal maturation is mediated by EOR-1/PLZF, MAU-2/cohesin loader, and SWI/SNF complex

**DOI:** 10.1038/s41598-018-26149-2

**Published:** 2018-05-21

**Authors:** Yoichi Shinkai, Masahiro Kuramochi, Motomichi Doi

**Affiliations:** 1 Molecular Neurobiology Research Group and DAI-Lab, Biomedical Research Institute, National Institute of Advanced Industrial Science and Technology (AIST), Central 6, 1-1-1, Higashi, Tsukuba, Ibaraki 305-8566 Japan; 2 Present Address: Graduate School of Frontier Sciences, The University of Tokyo, 5-1-5 Kashiwanoha, Kashiwa City, Chiba 277-8561 Japan; 3 AIST-UTokyo Advanced Operando-Measurement Technology Open Innovation Laboratory (OPERANDO-OIL), National Institute of Advanced Industrial Science and Technology (AIST), Chiba, 277-8565 Japan

## Abstract

Newborn neurons mature by distinct and sequential steps through the timely induction of specific gene expression programs in concert with epigenetic changes. However, it has been difficult to investigate the relationship between gene expression and epigenetic changes at a single-cell resolution during neuronal maturation. In this study, we investigated the maturation of hermaphrodite-specific neurons (HSNs) in *C. elegans*, which provided the link between chromatin dynamics, gene expression, and the degree of neuronal maturation at a single-cell resolution. Our results demonstrated that chromatin composition in the promoter region of several genes acting for neuronal terminal maturation was modulated at an early developmental stage, and is dependent on the function of the transcription factor EOR-1/PLZF and the cohesin loader MAU-2/MAU2. Components of the SWI/SNF chromatin remodeling complex were also required for the proper expression of terminal maturation genes. Epistasis analyses suggested that *eor-1* functions with *mau-2* and *swsn-1* in the same genetic pathway to regulate the maturation of HSNs. Collectively, our study provides a novel approach to analyze neuronal maturation and proposes that predefined epigenetic modifications, mediated by EOR-1, MAU-2, and the SWI/SNF complex, are important for the preparation of future gene expression programs in neuronal terminal maturation.

## Introduction

Chromatin structures are dynamically regulated by DNA methylation, histone post-translational modification, and nucleosome remodeling in order to induce proper gene expression programs throughout development. ATP-dependent chromatin remodeling enzymes alter chromatin accessibility by mediating the disassembly of nucleosomes and the exchange of histone variants^1^. Growing evidence has shown that nucleosome remodeling is involved in neurodevelopmental disorders and is the fundamental means for regulating gene expression programs during brain development^2–5^.

Post-mitotic neurons mature through distinct and sequential cellular steps including migration, neurite elongation, synapse formation, and the functional maturation of membrane potential. Each step requires the expression of specific genes in concert with epigenetic changes. For example, post-mitotic cerebellar granule neurons upregulate the expression of Grin2c, which is required for mature synaptic functions, in association with an increase in chromatin accessibility at transcriptional regulatory regions^6^. In contrast, genes acting during early neuronal development such as axon outgrowth are transiently upregulated at that period and show a decline in chromatin accessibility at later stage^6,7^. These reports underscore the importance of temporally controlled epigenetic alterations for gene expression during neuronal maturation. Nevertheless, the majority of chromatin accessibility changes have already occurred in immature newborn neurons and they remain static until the terminal maturation step^6,8^, raising the question of whether such pre-defined chromatin changes affect gene expression during neuronal terminal maturation. However, it has been difficult to define the relationship between gene expression and the underlying chromatin alterations within a heterogeneous cellular population including various developmental stages of neurons.

To avoid the difficulties caused by heterogeneity in a cellular population, we used hermaphrodite-specific neurons (HSNs) in the nematode *Caenorhabditis elegans*. In these hermaphrodite worms, HSNs are born from neuroblast cells at the tail and migrate to the mid-body region during embryogenesis, and the migration is completed by the L1 larval stage. Axon outgrowth initiates at the L3 larval stage. Finally, HSNs change their membrane and transmission properties by expressing genes required for functional maturation from the L4 larval stage onward, and to regulate egg-laying behavior at the adult stage^9^. This dynamic but slow maturation of HSNs and the transparent body of *C. elegans* allow easy access to gene expression activity and the chromatin state of each developmental stage by using extra-chromosomal reporter arrays^10,11^. These features provide a powerful means for analyzing the interplay between transcriptional activities and chromatin modifications during neuronal maturation at the single-neuron level.

To modify the chromatin states, chromatin remodeling complexes can be targeted to specific genomic loci by interacting with sequence-specific transcription factors^12–14^. Various transcription factors involved in the development of HSNs have been identified, including EOR-1, the *C. elegans* homolog of the Promyelocytic Leukemia Zinc Finger (PLZF)^15^. A recent study revealed that EOR-1 may regulate chromatin accessibility at transcriptional regulatory regions during the worm development^16^. Here, we show that EOR-1 affects chromatin dynamics in immature HSNs at the L1 larval stage and cell-autonomously enhances the later expression of genes required for HSN terminal maturation in adult hermaphrodite worms. We also showed that the core component of the SWI/SNF chromatin remodeling complex SWSN-1 and the cohesin loader subunit MAU-2 cell-autonomously regulate the expression of reporters for several genes acting for HSN maturation. Moreover, genetic analyses revealed that *eor-1* functions with *mau-2* and *swsn-1* in the same genetic pathway, suggesting previously unknown interactions between PLZF and the SWI/SNF and cohesin loader complexes during neuronal development. Our results provide a unique approach to analyze the relationship between chromatin alterations and gene expression during neuronal development and insight into the neurological mechanisms of neurodevelopmental disorders.

## Results

### *eor-1* regulates the expression of genes required for the HSN terminal maturation

**Figure 1:**
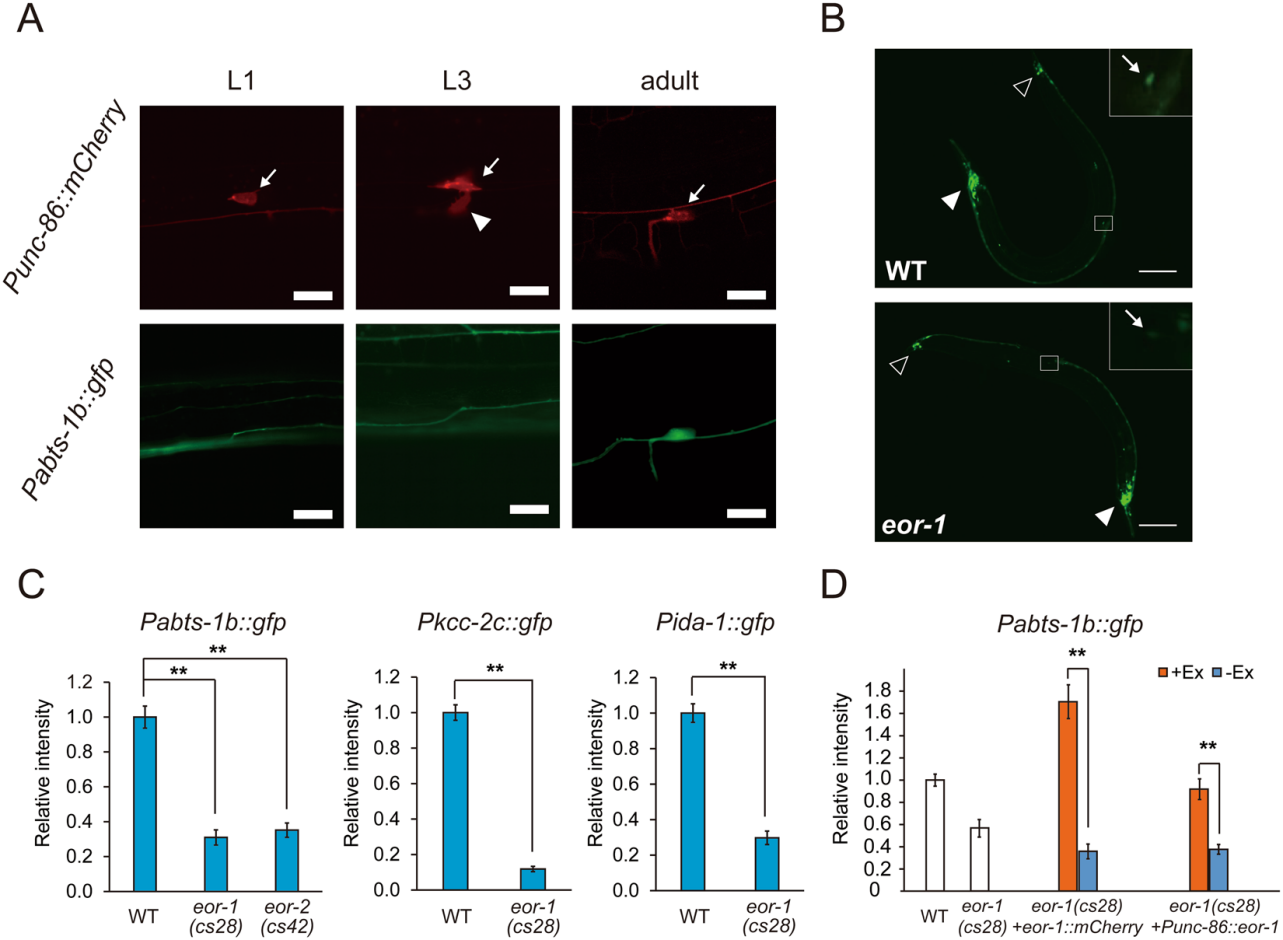
EOR-1 cell-autonomously regulates the expression of multiple genes required for HSN terminal maturation. (**A**) The maturation of HSNs is defined by developmental stages. *Punc-86::myr mCherry* marks both immature and mature HSNs (arrow). At the L3 larval stage, HSNs start to elongate a neurite (arrowhead). At the L4 to adult stages, the expression of *Pabts-1b::gfp* is gradually observed as HSNs mature. White scale bar indicates 10 µm. (**B**) Comparison of *Pabts-1b::gfp* expression at the adult stage between wild-type worms and *eor-1(cs28)* mutants. The HSN cell body is indicated by an arrow (inset). An open arrowhead indicates tail neurons and a closed arrowhead indicates head neurons. White scale bar indicates 100 µm. (**C**) Quantitative measurements of fluorescent intensity against *Pabts-1b::gfp*, *Pkcc-2c::gfp*, and *Pida-1::gfp* in HSNs at the adult stage. Error bars indicate SEM (n ≥ 30, **p < 0.01). (**D**) Fluorescent intensity of *Pabts-1b::gfp* in HSNs was analyzed in *eor-1(cs28)* mutants expressing EOR-1 by its own promoter or the *unc-86* promoter. Worms carrying the transgene (+Ex) and worms not carrying the transgene (−Ex) were compared. Error bars indicate SEM (n ≥ 30, **p < 0.01).

Transcription factors have been reported to regulate the target specificity of epigenetic enzymes in various organisms^13,17,18^, and the development of HSNs in *C. elegans*^19,20^. To uncover the mechanisms that govern gene expression and the underlying chromatin dynamics in developing HSNs, we performed a candidate screen for transcription factors that affect gene expression of HSNs in the adult hermaphrodite worms. HSNs express genes required for their terminal maturation at the L4 stage onward, such as *abts-1*, *kcc-2*, and *ida-1*, which encodes a sodium-driven chloride bicarbonate exchanger, a sodium chloride co-transporter, and a protein tyrosine phosphatase-like receptor, respectively^9,21^ (Fig. 1A). We found that *eor-1(cs28)* null mutants show decreased expression of GFP, which is under the control of the *abts-1b* promoter, in HSNs (Fig. 1B,C). On the other hand, *eor-1(cs28)* mutation did not appear to affect the expression of GFP, which is under the control of the same *abts-1b* promoter, in both head and tail neurons (Fig. 1B). Mutants in *eor-2*, which encodes a binding partner of EOR-1^22^, also showed decreased expression of GFP in HSNs (Fig. 1C). The phenotype observed in *eor-1* and *eor-2* mutants may be explained by the defect in the specific regulation of *abts-1* gene expression or a defect in the maturation of HSNs. To distinguish between these possibilities, we analyzed the expression of *kcc-2* and *ida-1*, which are also expressed from the L4 larval stage onward and required for the functional maturation of HSNs^21,23^. We found that *eor-1(cs28)* mutants show decreased expression of GFP in HSNs under the control of each promoter (Fig. 1C). Furthermore, *eor-1(cs28)* mutants were reported to show a defect in egg-laying, which is controlled by mature HSNs^24^. The defect of *eor-1(cs28)* mutants in the expression of *Pabts-1b::gfp* was rescued by the genomic *eor-1* DNA fragment fused with *mCherry*, *eor-1::mCherry* (Fig. 1D). On the other hand, unlike other transcription factors regulating HSN maturation steps^19,20,25,26^, the *eor-1* mutation did not affect migration or neurite elongation of HSNs (Fig. 1B, Supplementary Fig. S1). These results indicate that *eor-1* regulates the terminal maturation of HSNs by modulating expression of multiple genes required for the maturation, rather than indirectly through earlier deficits in HSN development.

### EOR-1 functions in HSNs

Neurite elongation of HSNs has been shown to be regulated by FGF signaling derived from vulval epithelium cells^27^. This suggests that the maturation of HSNs may be affected by surrounding cells. However, mutations in *egl-15*, a gene encoding the sole *C. elegans* FGF receptor, did not affect the expression of *Pabts-1b::gfp* in HSN neurons (Supplementary Fig. S2), raising the possibility that *eor-1* plays a cell-autonomous role in the maturation of HSNs. To determine whether *eor-1* cell-autonomously functions to regulate the maturation of HSNs, we generated transgenic *eor-1(cs28)* mutants expressing EOR-1 under the control of the *unc-86* promoter, which drives gene expression in multiple neurons including HSNs from embryogenesis onward, but not in surrounding cells. The *unc-86* promoter has previously been used to examine the cell-autonomous function of genes in HSNs^28,29^. The expression of EOR-1 under the control of *unc-86* promoter recovered the phenotype of *eor-1(cs28)* mutants, and the transgenic *eor-1(cs28)* mutants showed almost the same expression of *Pabts-1b::gfp* in HSNs as wild-type animals (Fig. 1D). However, other phenotypes, such as uncoordinated movement and the larval lethality of *eor-1(cs28)* mutants, did not recover with the expression of EOR-1 under the control of the *unc-86* promoter. These results suggested that the expression of EOR-1 in specific neurons is sufficient for the normal expression of *Pabts-1b::gfp* in HSNs.

### EOR-1 binds to *abts-1* promoter regions

**Figure 2:**
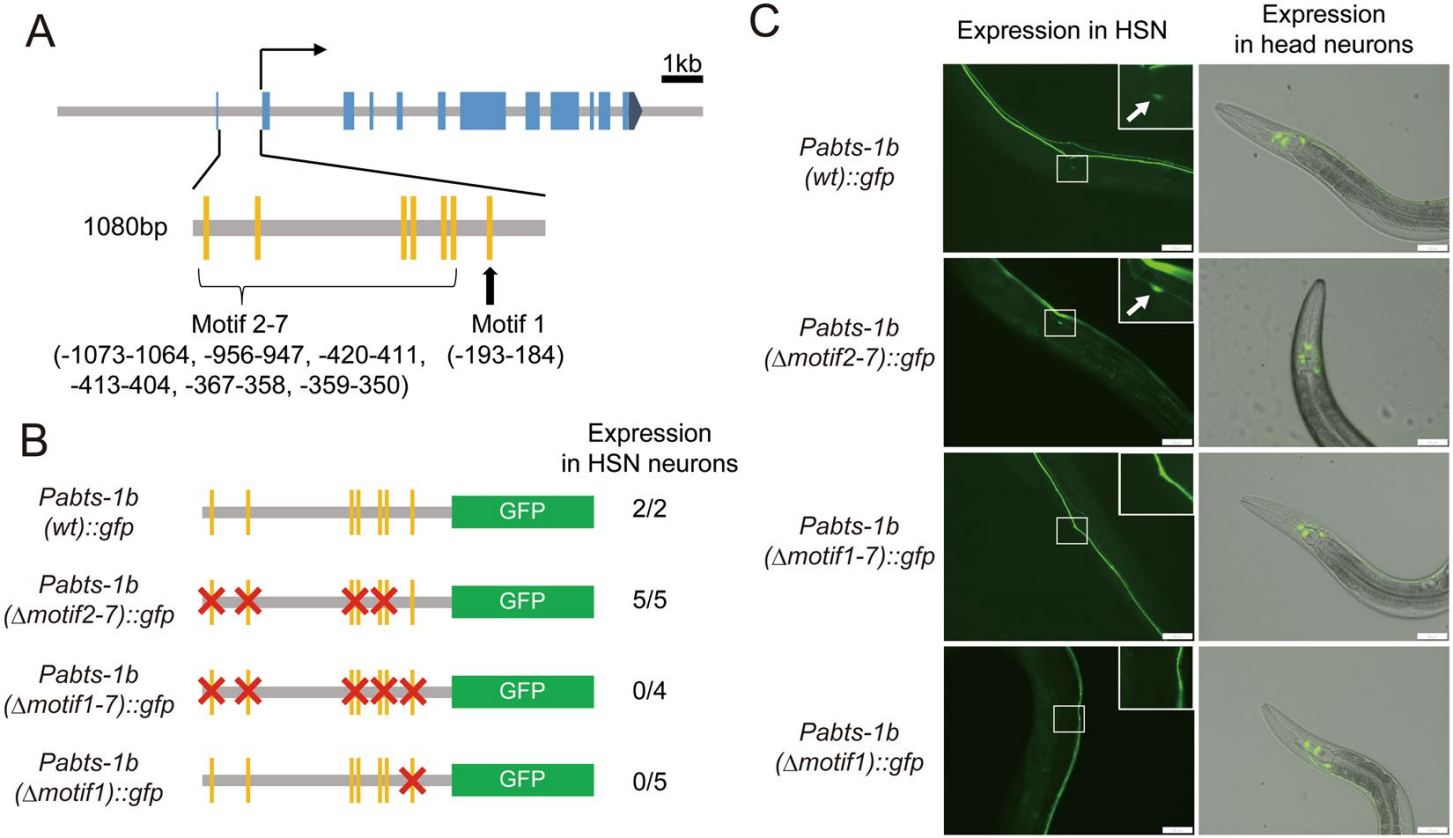
EOR-1 binds to the regulatory region of *abts-1* gene required for HSN terminal maturation. (**A**) The gene structure of the *abts-1* gene and putative EOR-1 binding motifs within the 1.1 kb promoter of the *abts-1b* isoform. Blue boxes indicate exons of the *abts-1* gene. The *abts-1b* gene initiates transcription from the second exon. Vertical yellow lines indicate putative EOR-1 binding motifs. (**B**) Mutation analysis of the putative EOR-1 binding motif for the expression of *Pabts-1b::gfp* in HSNs. The number of lines expressing gfp in HSNs are shown in the total number of lines. (**C**) Representative examples for mutation analysis of the putative EOR-1 binding motif for the expression of *Pabts-1b::gfp* in HSNs and head neurons. The HSN cell body is indicated by an arrow (inset). White scale bar indicates 50 µm.

*eor-1* encodes the zinc-finger transcription factor homologous to human PLZF. The binding consensus sequence of EOR-1 has been previously obtained from the analysis of ChIP-Seq data for EOR-1^30^. To test whether EOR-1 directly regulates the expression of *Pabts-1b::gfp*, we analyzed the existence of the binding consensus sequence of EOR-1 in the *abts-1b* promoter. We found a fully identical motif (motif 1: GAGACGCAGA) to the EOR-1 binding consensus sequence at about 200 bp upstream of the start codon of *abts-1b*. Furthermore, according to the JASPAR database, we found six additional motifs (motif 2−7) similar to the EOR-1 binding consensus sequence in the *abts-1b* promoter (Fig. 2A). To characterize the importance of each EOR-1 binding motif, we compared the activity of the *abts-1b* promoter by mutating each motif. Simultaneous mutation of all seven motifs eliminated expression in HSNs, while expression in head neurons was not affected (Fig. 2B,C). These results are consistent with the expression pattern of *Pabts-1b::gfp* of *eor-1(cs28)* mutants. Next, we searched the motif sufficient for the expression in HSNs. When we mutated the fully identical motif (motif 1) to the EOR-1 binding consensus sequence at about 200 bp upstream of the start codon of *abts-1b*, expression in HSNs disappeared. In contrast, mutating the six additional motifs (motif 2–7) did not eliminate expression in HSNs, suggesting that the motif 1 at about 200 bp upstream of the start codon is required and sufficient for the expression of *Pabts-1b::gfp* in HSNs. As mentioned above, the expression of *Pabts-1b::gfp* in HSNs was *eor-1*-dependent (Fig. 1B,C). Moreover, analysis of ChIP-Seq data for EOR-1 from modENCODE indicated that EOR-1 was localized among the *abts-1b* promoter (Supplementary Fig. S3). Therefore, these findings suggested that EOR-1 directly regulates the expression of *abts-1* in HSNs by binding to the promoter region. Analysis of ChIP-Seq data for EOR-1 also indicated that EOR-1 was localized to the *kcc-2* and *ida-1* promoters, in addition to the *abts-1* promoter (Supplementary Fig. S3), suggesting the broad role of EOR-1 in regulating the expression of genes involved in the maturation of HSNs.

### Expression of EOR-1 at an early developmental stage is sufficient for normal HSN maturation

**Figure 3:**
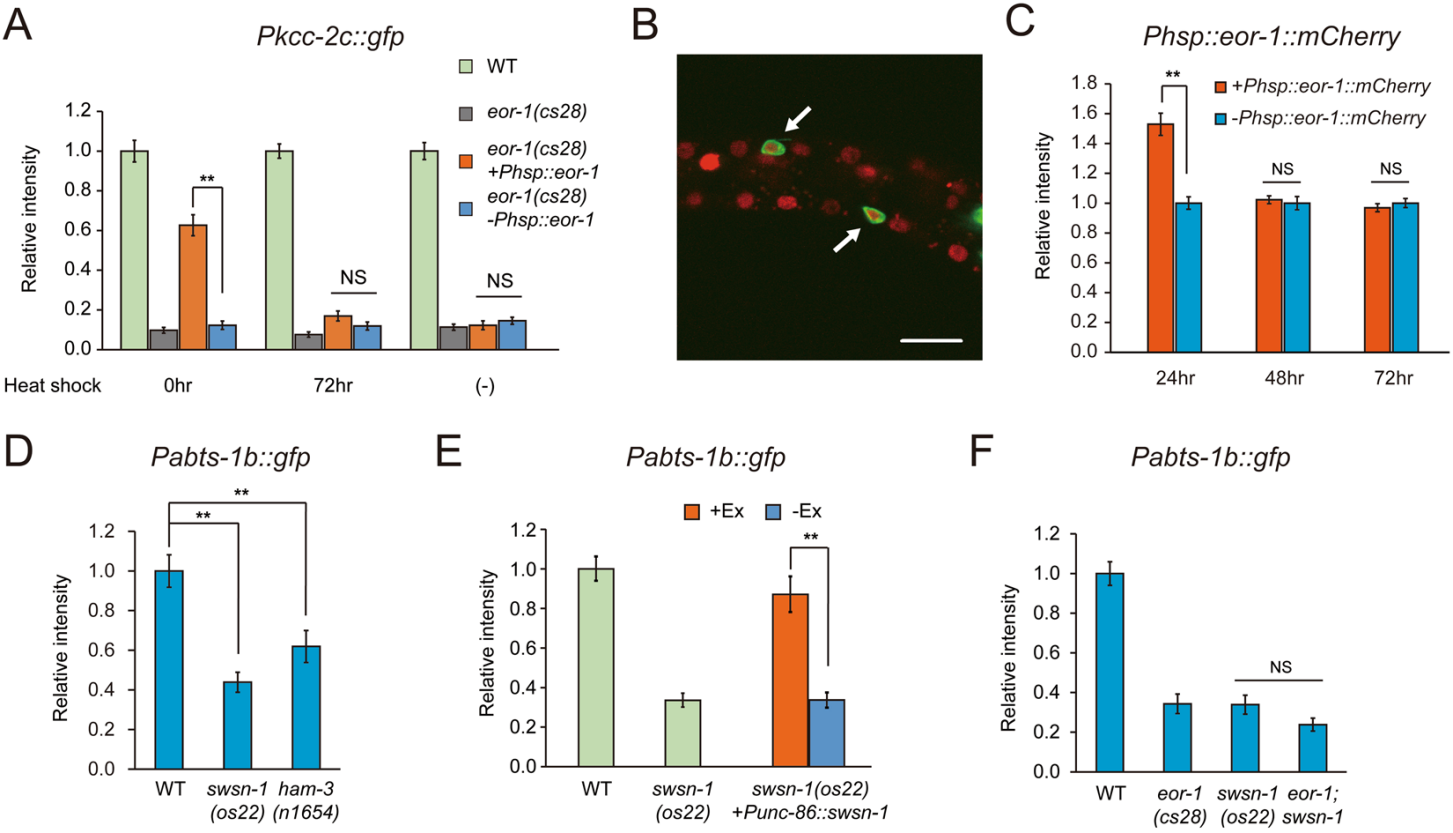
Chromatin remodeling affects the gene expression during HSN terminal maturation. (**A**) Fluorescent intensity of *Pkcc-2c::gfp* in HSNs was analyzed in *eor-1(cs28)* mutants carrying *Phsp-16.2::eor-1* after heat-shock. Heat-shock was delivered to eggs (0 h) or worms at the L4 larval stage to young adult stage (72 h). Worms carrying the transgene (+Ex) and worms not carrying the transgene (−Ex) were compared at the adult stage (96 h). Error bars indicate SEM (n ≥ 30, **p < 0.01). (**B**) The expression of genomic *eor-1::mCherry* in HSNs at the L1 larval stage. *Punc-86::myr gfp* marks HSNs (arrow). White scale bar indicates 10 µm. (**C**) Fluorescent intensity of *Phsp-16.2::eor-1::mCherry* in HSNs was analyzed in WT worms at 24 hr (L1), 48 hr (L2/L3), and 72 hr (L4/young adult) after heat-shock. Worms carrying the transgene (+Ex) and worms not carrying the transgene (−Ex) were compared. Error bars indicate SEM (n ≥ 28, **p < 0.01). (**D**) Comparison of *Pabts-1b::gfp* expression in HSNs at the adult stage between wild-type worms, *swsn-1(os22)* mutants, and *ham-3(n1654)* mutants. Error bars indicate SEM (n ≥ 34, **p < 0.01). (**E**) *Pabts-1b::gfp* in HSNs was analyzed in *swsn-1(os22)* mutants expressing SWSN-1 by the *unc-86* promoter. Worms carrying the transgene (+Ex) and worms not carrying the transgene (−Ex) were compared. Error bars indicate SEM (n ≥ 36, **p < 0.01). (**F**) The genetic interaction between *eor-1* and *swsn-1* in *Pabts-1b::gfp* expression at the adult stage. Error bars indicate SEM (n ≥ 30).

Transcription of genes involved in the maturation of HSNs begins at the L4 larval stage. To determine when EOR-1 functions to regulate the expression of genes required for the maturation of HSNs, we utilized worms that express EOR-1 under the control of a heat shock promoter. We delivered a 2h-heat shock to the worms soon after egg-laying (0 h) or at 72 h after egg-laying, which corresponds to the gastrulation stage of embryogenesis and the developmental stage L4 to young adult, respectively. Surprisingly, the expression of EOR-1 at 72 h after egg-laying did not recover the expression of *Pkcc-2c::gfp* in *eor-1(cs28)* mutants, while the expression of EOR-1 soon after egg-laying did rescue the defect (Fig. 3A). Combined with the previous data showing that EOR-1 functions at ~470 min after first cell division during embryogenesis to induce male-specific apoptosis of HSNs^15,31^, these results motivated us to analyze the temporal expression pattern of *eor-1*. The *eor-1::mCherry* that contains *eor-1*-rescuing activity for the terminal maturation showed broad mCherry expression starting at embryogenesis (Supplementary Fig. S4). We confirmed the nuclear localization of EOR-1::mCherry in HSNs, visualized by myristoyl-tagged GFP under the control of the *unc-86* promoter, at the L1 larval stage of wild-type worms (Fig. 3B). These results suggested that EOR-1 may function to regulate the expression of genes required for the maturation of HSNs at an early developmental stage, when expression of the genes is not yet observed. However, it could be possible that the EOR-1 proteins expressed at L1 larval stage are stable during developmental stages and can affect the gene expression required for the maturation of HSNs at L4 larval stage. To rule out the possibility, we analyzed the time course of degradation of EOR-1::mCherry expressed soon after egg-laying in HSNs under the control of a heat shock promoter (Fig. 3C). The amount of EOR-1::mCherry protein was significantly abundant at 24 hr after heat shock, which corresponds to the L1 stage. However, the mCherry signal in HSNs of transgenic animals was reduced to the background levels observed in non-transgenic animals at 48 hr and 72 hr after heat shock, which correspond to the L2/L3 and L4/young adult stage, respectively. These results reinforced the significance of EOR-1 expressed at an early developmental stage for the later gene expression during HSN maturation.

### *swsn-1* is required for the maturation of HSNs

Our results suggest that the effect of EOR-1 expression at an early developmental stage lasts until the adult stage. Combined with a recent report showing that *eor-1* may regulate chromatin accessibility dynamics at transcriptional regulatory regions during development^16^, this fact implies that EOR-1 regulates gene expression by modulating the epigenetic state of the genes expressed during the maturation step of HSNs. The SWI/SNF chromatin remodeling complex has been reported to regulate the maturation process of HSNs^23^, and serves to alter the structure of nucleosomes in order to regulate transcriptional activity^32^. To test whether the SWI/SNF chromatin remodeling complex affects the expression of genes required for the maturation of HSNs, we analyzed the expression of *Pabts-1b*::*gfp* in HSNs of *swsn-1(os22)* mutants and *ham-3(n1654)* mutants, both of which have been reported to show egg-laying defects^23,33^. *swsn-1* and *ham-3* encode the *C. elegans* homolog of human SMARCC1 and human SMARCD, respectively. The encoded proteins are subunits of the SWI/SNF chromatin remodeling complex. *swsn-1* has been shown to be ubiquitously expressed and localized to nuclei^33^. *swsn-1(os22)* and *ham-3(n1654)* mutants showed decreased expression of *Pabts-1b::gfp* in HSNs (Fig. 3D). To determine whether *swsn-1* functions to regulate the expression of *Pabts-1b::gfp* cell-autonomously, we expressed SWSN-1 in HSNs under the control of the *unc-86* promoter. *swsn-1(os22)* mutants expressing SWSN-1 in HSNs showed nearly the same expression of *Pabts-1b::gfp* as wild-type (Fig. 3E). Therefore, *swsn-1* may regulate *Pabts-1b::gfp* expression in HSNs cell-autonomously. These data imply that chromatin remodeling complexes directly or indirectly regulate the expression of *Pabts-1b::gfp* in HSNs.

### SWSN-1 interacts with EOR-1 to regulate HSN maturation

EOR-1 was shown to genetically interact with SWI/SNF chromatin remodeling complex in a systematic analysis^34^. Furthermore, previous ChIP-seq analyses suggested that SWSN-1 is highly enriched around EOR-1 binding motifs^18,30^. As *swsn-1* and *eor-1* function in the same neurons to regulate HSN maturation, these may act in the same genetic pathway. To analyze the genetic relationship between them, we attempted to create *eor-1;swsn-1* double-mutant animals. However, we could not obtain double mutants due to apparent lethality. Since the lethality observed in *eor-1(cs28)* null mutants was rescued by maternal expression of EOR-1^24^, we employed homozygous *eor-1(cs28);swsn-1(os22)* offspring from heterozygous *eor-1(cs28)/*+*;swsn-1(os22)* animals. First, we analyzed the maternal effect of EOR-1 on the maturation of HSNs. Homozygous *eor-1(cs28)* offspring from heterozygous *eor-1(cs28)*/+ animals showed a defect in the expression of *Pabts-1b::gfp* in HSNs (Supplementary Fig. S5), indicating that maternal EOR-1 expression was not sufficient for the maturation of HSNs. This maternal insufficiency for HSN maturation enabled us to analyze the genetic interaction between *eor-1* and *swsn-1*. In addition, homozygous *eor-1(cs28);swsn-1(os22)* offspring from heterozygous *eor-1(cs28)/*+;*swsn-1(os22)* animals were viable. The homozygous *eor-1(cs28);swsn-1(os22)* offspring showed nearly the same phenotype as homozygous *eor-1(cs28)* offspring in the HSN expression of *Pabts-1b::gfp* (Fig. 3F). Therefore, *eor-1* and *swsn-1* may function in the same genetic pathway.

### H2A.Z is localized to the *abts-1b* promoter at the L1 to L2 early larval stage

**Figure 4:**
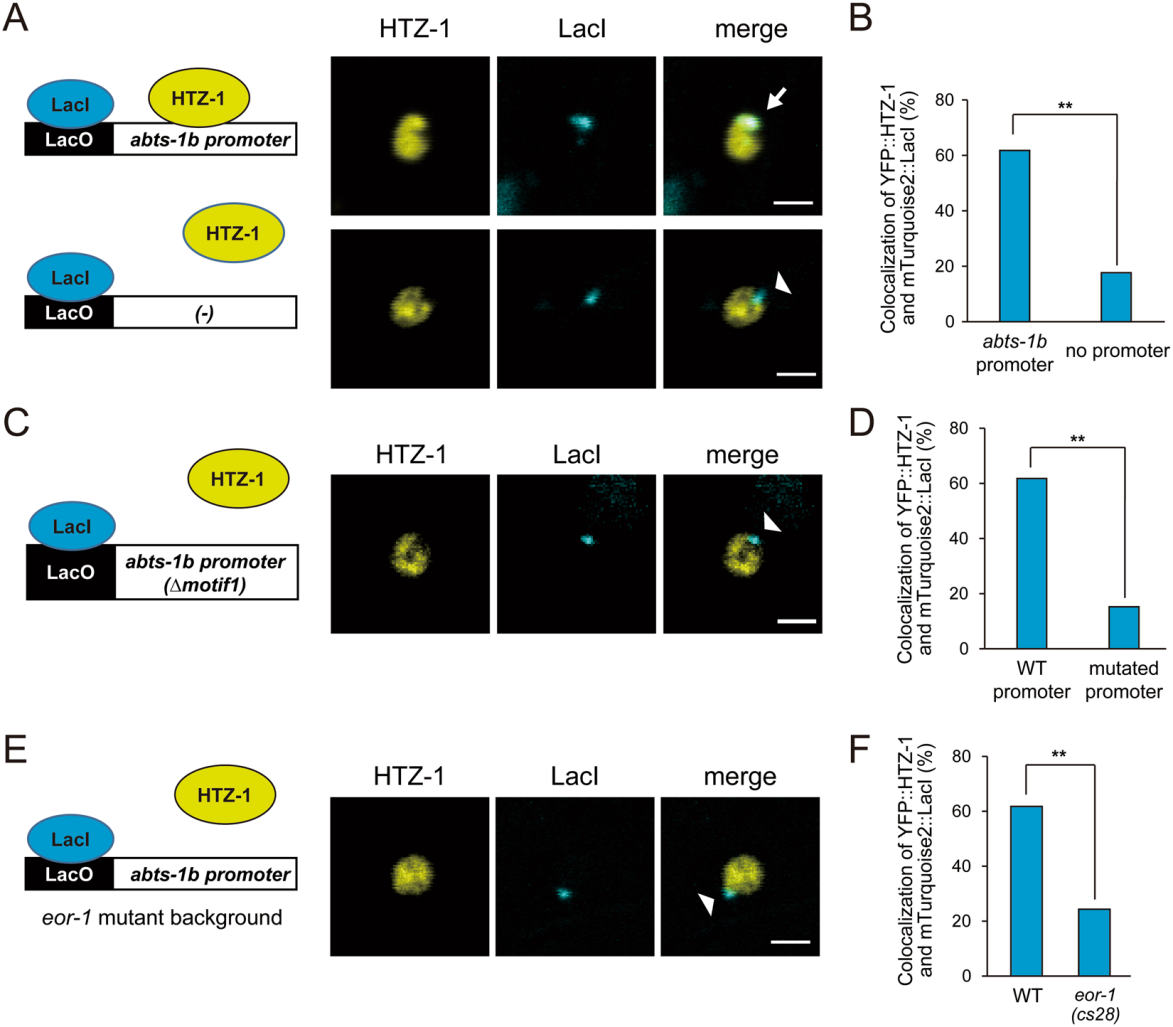
EOR-1-dependent localization of the histone variant HTZ-1 at the regulatory region of *abts-1b*. (**A**) Localization of YFP::HTZ-1 and extrachromosomal LacO reporter arrays carrying the *abts-1b* promoter or no promoter in HSNs at the L1 to L2 larval stage. The extrachromosomal LacO reporter array was visualized by the binding of mTurquoise2::LacI. Arrow indicates co-localization of YFP::HTZ-1 and reporter arrays. Arrowhead indicates exclusion of YFP::HTZ-1 from reporter arrays. White scale bars show 2 µm. (**B**) Quantitative data from the co-localization of YFP::HTZ-1 and reporter arrays carrying the *abts-1b* promoter or no promoter. The percentage of the co-localization was analyzed. n ≥ 34, **p < 0.01. (**C**) Localization of YFP::HTZ-1 and extrachromosomal LacO reporter arrays carrying the mutated *abts-1b* promoter (Δmotif1) at the L1-2 larval stages in HSNs of wild-type worms. The extrachromosomal LacO reporter array was visualized by the binding of mTurquoise2::LacI. Arrowheads indicate exclusion of YFP::HTZ-1 from reporter arrays. White scale bar shows 2 µm. (**D**) Quantitative data from the co-localization of YFP::HTZ-1 and reporter arrays carrying the *abts-1b* promoter or the mutated *abts-1b* promoter (Δmotif1). The percentage of the co-localization was analyzed. n ≥ 33, **p < 0.01. (**E**) Localization of YFP::HTZ-1 and extrachromosomal LacO reporter arrays carrying the wild-type *abts-1b* promoter at the L1-2 larval stages in HSNs of *eor-1(cs28)*. Arrowheads indicate exclusion of YFP::HTZ-1 from reporter arrays. White scale bar shows 2 µm. (**F**) Percentages of the co-localization of YFP::HTZ-1 and reporter arrays carrying the *abts-1b* promoter were compared between the wild-type background and *eor-1(cs28)* mutant background. n ≥ 34, **p < 0.01.

The SWI/SNF chromatin remodeling complex affects the composition of histone H2A.Z in nucleosomes at the target loci^35,36^. The accumulation of H2A.Z in promoter regions correlates with the activity of gene expression and may be a landmark for nucleosome-free regions in active promoters^37,38^. Therefore, we hypothesized that H2A.Z may be deposited in the *abts-1b* promoter region during HSN maturation. To examine the localization of H2A.Z in the *abts-1b* promoter region, we visualized HTZ-1, the *C. elegans* homolog of H2A.Z, and extra-chromosomal arrays containing *abts-1b* promoter sequences by using Nuclear spot assays^39^. We generated transgenic worms expressing both YFP::HTZ-1 and mTurquoise2::LacI, which bind to Lac operator sequences (LacO). Then, the extra-chromosomal array (reporter array) that contains multiple copies of LacO repeats and *abts-1b* promoter sequences was introduced into the transgenic worms. The reporter array in nuclei can be visualized by the binding of mTurquoise2::LacI to LacO, indicating the localization of exogenous *abts-1b* promoter sequences. To analyze whether HTZ-1 was deposited to the *abts-1b* promoter, we examined the co-localization of mTurquoise2::LacI and YFP::HTZ-1 in the nucleus of HSNs. We observed frequent co-localization of mTurquoise2::LacI and YFP::HTZ-1 in HSNs of L1 to L2 larval stage worms with the reporter array carrying exogenous *abts-1b* promoter sequences (Fig. 4A). The frequency was significantly higher than that of worms with reporter arrays that contained LacO repeats but not the *abts-1b* promoter (Fig. 4B). These results suggested that HTZ-1 was deposited to the *abts-1b* promoter in HSNs at the L1 to L2 larval stage, supporting the possibility that the *abts-1b* promoter directly receives chromatin remodeling at an early developmental stage in order to induce later gene expression starting from the L4 stage. Such frequent co-localization of HTZ-1 and the *abts-1b* promoter was attenuated at the L4 larval stage (Supplementary Fig. S6A,B). These results are consistent with the widely reported concept that H2A.Z dissociates from chromatin once transcription is initiated^40–43^. Furthermore, the reporter array with *abts-1b* promoter sequences was localized to the nuclear periphery at the L1 larval stage and apparently changed the sub-nuclear localization from periphery to center during development (Supplementary Fig. S6A). In general, heterochromatin regions and euchromatin regions are localized to the nuclear periphery and the center of nuclei, respectively. Therefore, the reporter array likely recapitulated proper chromatin modifications *in vivo*.

### Necessity of *eor-1* in the localization of H2A.Z in the *abts-1b* promoter

Next, we analyzed whether *eor-1* is required for the deposition of HTZ-1 to the *abts-1b* promoter. We generated transgenic lines that contained reporter arrays carrying the *abts-1b* promoter with a mutated EOR-1 binding motif (Δmotif1, Fig. 2B). The transgenic lines showed a significant decrease in the frequency of the co-localization of mTurquoise2::LacI and YFP::HTZ-1 in the nucleus of HSNs at the L1 to L2 larval stage, compared with worms containing reporter arrays of the wild-type *abts-1b* promoter (Fig. 4C,D). Furthermore, in an *eor-1(cs28)* mutant background, the frequency of the co-localization of mTurquoise2::LacI and YFP::HTZ-1 significantly decreased on the wild-type *abts-1b* promoter (Fig. 4E,F). These results revealed that *eor-1* is required for the deposition of HTZ-1 to the *abts-1b* promoter region. The requirement of *eor-1* for the deposition of HTZ-1 at the L1 to L2 larval stage suggested that EOR-1 functions to regulate the expression of genes required for HSN maturation by modifying the epigenetic state of the genes at an early developmental stage.

### Cohesin loader MAU-2 affects the gene expression during HSN terminal maturation

**Figure 5:**
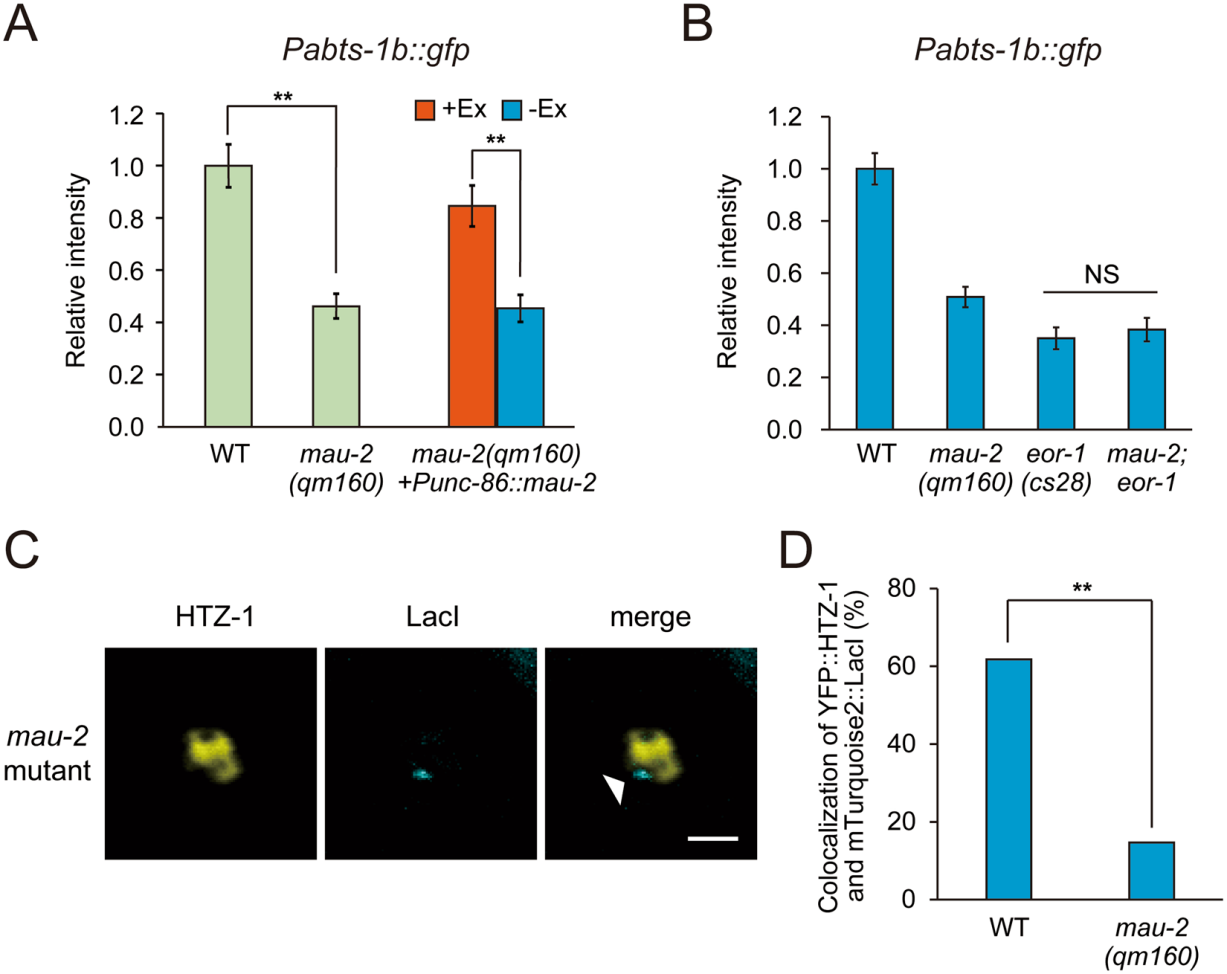
Cohesin loader MAU-2 affects the gene expression during HSN terminal maturation. (**A**) Fluorescent intensity of *Pabts-1b::gfp* in HSNs was analyzed in *mau-2(qm160)* mutants expressing MAU-2 by the *unc-86* promoter. Error bars indicate SEM. n ≥ 32, **p < 0.01. (**B**) The genetic interaction between *eor-1* and *mau-2* in *Pabts-1b::gfp* expression at the adult stage. Error bars indicate SEM (n ≥ 30). (**C**) Localization of YFP::HTZ-1 and extrachromosomal LacO reporter arrays carrying the *abts-1b* promoter at the L1-2 larval stages in HSNs of *mau-2(qm160)* mutants. The extrachromosomal LacO reporter array was visualized by the binding of mTurquoise2::LacI. Arrowhead indicates exclusion of YFP::HTZ-1 from reporter arrays. White scale bar shows 2 µm. (**D**) Percentage of co-localization of YFP::HTZ-1 and reporter arrays carrying the *abts-1b* promoter in HSNs at the L1 to L2 larval stage was compared between the wild-type background and *mau-2(qm160)* mutant background. n ≥ 34, **p < 0.01.

Our data suggested that epigenetic modifications occurred at an early developmental stage and lasted until later gene expression as an epigenetic memory. However, it is yet unclear how the epigenetic modifications could be established and be maintained during development. To get an insight into the molecular mechanism for the modification, we sought another mutant defective in the expression of genes required for HSN maturation. Among egg-laying defective mutants, we identified *mau-2(qm160)* null mutants, which showed common defects with *eor-1(cs28)* mutants. *eor-1(cs28)* and *mau-2(qm160)* mutants share multiple abnormalities such as egg-laying defects, uncoordinated movement, dye-filling defects, and rod-like lethality^24,44^. *mau-2* encodes a homolog of yeast Scc4, which loads cohesin rings to the target genomic loci. The Scc2/Scc4 cohesin loader binds to nucleosome-free genomic loci with active transcription states^45^. In this study, *mau-2(qm160)* mutants showed decreased expression of *Pabts-1b::gfp* in HSNs (Fig. 5A). Defects in the expression of *Pabts-1b::gfp* were rescued by the expression of MAU-2 under the control of the *unc-86* promoter. These results suggested that *mau-2* functions to regulate the expression of genes required for the maturation of HSNs cell-autonomously.

The phenotypic similarity between *eor-1* and *mau-2* implied the possibility that these molecules could act in the same genetic pathway. To analyze the genetic interaction, we examined the phenotype of *mau-2(qm160);eor-1(cs28)* double null mutant animals. To avoid lethality, we employed homozygous *mau-2(qm160);eor-1(cs28)* offspring from heterozygous *mau-2(qm160);eor-1(cs28)/*+ animals. The homozygous *mau-2(qm160);eor-1(cs28)* offspring showed nearly the same phenotype as homozygous *eor-1(cs28)* offspring in the expression of *Pabts-1b::gfp* in HSNs (Fig. 5B). Therefore, *eor-1* and *mau-2* may function in the same genetic pathway.

To obtain mechanistic insight into how *mau-2* regulates gene expression, we analyzed the localization of YFP::HTZ-1 in HSNs in *mau-2(qm160)* mutants. If MAU-2 acts only for the maintenance of the epigenetic modification rather than the establishment, YFP::HTZ-1 is assumed to properly localize in HSNs at an early developmental stage, and the localization should be disturbed as development proceeds. Unexpectedly, the *mau-2(qm160)* mutation significantly decreased the frequency of the co-localization of mTurquoise2::LacI and YFP::HTZ-1 in HSNs at the L1 to L2 larval stage (Fig. 5C,D). These results are consistent with the idea that *mau-2* and *eor-1* cooperate to regulate gene expression by establishing chromatin features in HSNs at an early developmental stage.

In conclusion, *eor-1* may act at an early developmental stage to regulate the expression of genes in later stages of HSN maturation, and this process is cooperative with chromatin remodeling complexes and cohesin loaders.

## Discussion

The maturation of post-mitotic neurons consists of multiple processes that require temporally controlled gene expression associated with chromatin structure changes. To gain mechanistic insight into chromatin regulation during neuronal maturation, we studied gene expression during late stages of neuronal maturation at a single-neuron level by linking the degree of neuronal maturation with the chromatin state of the promoter region. Our study revealed that *eor-1* regulates both the chromatin state and gene expression, which mediate the late stages of HSN maturation. Importantly, such chromatin regulation by *eor-1* occurs at an early developmental stage, when the expression of genes that mediate the late maturation of HSN neurons is not observed. Our heat-shock rescuing experiment implies the critical period for the function of EOR-1, since the expression of EOR-1 at a later developmental stage could not recover the phenotype of *eor-1(cs28)* mutants. This observation is consistent with the potential model that EOR-1-dependent chromatin remodeling at the promoter region is a prerequisite for the later activation of gene expression (Supplementary Fig. S7). Although further studies such as the development of single-cell type ChIP-seq will be needed to compellingly support this model, our study demonstrates an alternative approach to analyze the relationship between gene expression and chromatin alterations during neuronal maturation.

*eor-1* encodes a zinc finger transcription factor homologous to human PLZF. PLZF exerts various biological functions during the development of multiple tissues^46^. Although PLZF is expressed in a temporally dynamic pattern in the developing CNS, and functions to maintain neuronal progenitors in neurogenesis^47,48^, the role of PLZF in the terminal maturation of neurons is poorly understood. Our single-cell analysis indicated that *eor-1* mutation did not eliminate the expression of genes for terminal maturation but rather decreased the expression. Furthermore, the extension of a neurite from HSNs was observed in *eor-1* mutants. These observations suggest that *eor-1* mutation did not affect the determination of HSN cell fate. Therefore, we propose that *eor-1* functions to enhance lineage specific gene expression of HSNs by modulating the chromatin state of the specific genes. The function of EOR-1 in HSN maturation may be analogous to that of PLZF in the differentiation of mammalian stem cells.

In this study, we also showed that the components of the SWI/SNF chromatin remodeling complex such as SWSN-1/SMARCC1 and HAM-3/SMARCD are involved in the terminal maturation of HSNs. Furthermore, epistasis analysis suggested EOR-1 genetically interacted with SWSN-1. Interestingly, previous ChIP-seq analyses reported that two components of the SWI/SNF complex is highly enriched in a DNA motif nearly identical to the binding consensus sequence of EOR-1 in *C. elegans*^18,30^. Chromatin remodeling complexes may be recruited to target sites by interacting with pioneer transcription factors, which can bind to target sequences on condensed nucleosomal DNA^49^. Here, we showed that *eor-1* is required for the chromatin remodeling on the *abts-1b* promoter region that contains the binding motif of EOR-1. Furthermore, recent ATAC-seq data showed that EOR-1 may regulate chromatin accessibility at transcriptional regulatory regions during development^16^. Although our genetic analysis has not shown the genetic hierarchy between *eor-1* and *swsn-1*, it is highly possible that EOR-1 may function as a pioneer transcription factor for recruiting SWI/SNF chromatin remodeling complexes to target sites.

The cohesin complex also plays an important role in chromatin architecture and transcriptional regulation^50^. Our results implied a functional interaction between the cohesin loader complex and SWI/SNF chromatin remodeling complexes. Such functional similarity between the two complexes has also been observed in HSN neuronal migration^23,44^. Indeed, the yeast Scc2/Scc4 cohesin loader complex, recruited by the RSC chromatin remodeling complex, functions to maintain nucleosome-free regions, although it is unknown how the RSC complex is targeted to specific genomic sites^45^. Therefore, we propose a potential model where the interactions between EOR-1, SWSN-1, and MAU-2 alter chromatin structure and chromatin composition at EOR-1 binding sites in order to prepare for subsequent gene expression programs.

Chromatin regulation and gene expression can be temporally disassociated in principle, since these events are performed by distinct machineries^51^. However, it is yet unclear why the “pre-defined” nucleosome alterations are required for future gene expression during development. Poised RNA polymerase II (Pol II) preferentially occupies the promoter region of genes that are dynamically regulated during development^52,53^. This poised Pol II occupancy prepares genes for future expression by establishing a permissive state that enables rapid and synchronous gene induction^54,55^. Interestingly, poised Pol II targeted to promoter regions by a pioneer transcription factor also helps nucleosome depletion and contributes to the accessibility of the promoters^56,57^. Hence, it is conceivable that poised Pol II and chromatin remodeling complexes recruited by a pioneer transcription factor have a cooperative function to prepare future gene expression during development. A recent report indicated that developmentally regulated genes generally lack canonical post-translational histone modifications^58^. Thus, it could be possible that “pre-defined” nucleosome remodeling may be an alternative pathway to dynamically regulate genes during development, and may help developmentally coordinated neuronal maturation by rapid and synchronous gene induction.

In this study, we showed that HTZ-1, the *C. elegans* homolog of H2A.Z, was transiently accumulated on the promoter region of a gene required for the functional maturation of HSNs during development. Interestingly, recent reports have revealed that H2A.Z accumulates specifically at promoter regions, where the occupancy of H2A.Z correlates with that of poised Pol II during *C. elegans* development, implying the role of H2A.Z in pausing Pol II and preparing for rapid and synchronous gene induction^59,60^. Consistently, our results demonstrated that H2A.Z deposition on *abts-1b* promoter was removed once transcription was initiated at the L4 stage, raising the possibility that H2A.Z deposition mediated by EOR-1 and MAU-2 affects the transcriptional activity of genes required for HSN maturation. Hence, the interactions between EOR-1, SWSN-1, and MAU-2 might coordinate neuronal maturation and gene expression through H2A.Z incorporation into the genes required for HSN maturation.

In summary, we report the significance and the molecular basis of chromatin alterations for neuronal maturation in *C. elegans*. A similar mechanism may operate in higher organisms, since the maturation of HSN neurons consists of typical processes observed in mammalian neurons including cell migration, neurite elongation, synapse formation, and the functional maturation of membrane potential. Importantly, the functional link between MAU-2, SWSN-1, and EOR-1 may explain overlapping features observed among human disorders. Cornelia de Lange syndrome, caused by abnormalities of the human cohesin loader complex, is in some cases difficult to distinguish from Coffin-Siris syndrome, a SWI/SNF chromatin remodeling complex disorder^61–63^. Furthermore, loss-of-function mutations in human PLZF cause phenotypes commonly observed in both syndromes^64^. Thus, it is possible that neurological abnormalities in these disorders arise from dysfunctions in common molecular pathways that affect chromatin structures and transcriptional regulation. Further analyses may provide insight on the molecular basis of temporally regulated gene expression programs through chromatin dynamics, and facilitate the molecular understanding of these serious human disorders.

## Materials and Methods

### Strains and culture

The strains used in this study were as follows:

Wild-type strain N2, *eor-1(cs28)*, *eor-1(ok1127)*, *eor-2(cs42)*, *swsn-1(os22)*, *ham-3(n1654)*, *mau-2(qm160)*, *egl-15(n484)*, CX5974 [*kyIs262* (*Punc-86::myr gfp, Podr-1::rfp*)], LX1514 (*lin-15(n765); vsIs138* [*Pabts-1b::gfp, lin15*(+)]) outcrossed to the wild-type strain to remove *lin-15(n765)*, BL5717 (*him-8(e1489); inIs179* [*Pida-1::gfp*]) outcrossed to the wild-type strain to remove *him-8(e1489)*, and VC2448 (*kcc-2(ok3074)/nT1* [*qIs51*]) outcrossed to the wild-type strain to remove *kcc-2(ok3074)*. All strains were cultured on NGM plates with the *E. coli* strain OP-50 under standard conditions^65^.

### DNA constructs and Germline transformation

For the mutation analysis of putative EOR-1 binding motifs in the *abts-1b* promoter, the *abts-1b* promoter (1.1 kb) and the *abts-1* 3′UTR (0.4 kb) were amplified by PCR from genomic DNA and were sub-cloned into the pPD95.75 vector to construct *Pabts-1b::gfp::abts-1* 3′UTR. The putative EOR-1 binding motif was mutated by substituting four nucleotides in the core sequence (WT: GAGAcgcaga» mutant: CCCCcgcaga). For the generation of the insertion line expressing *Pkcc-2c::gfp::kcc-2* 3′UTR, the *kcc-2c* promoter (7.9 kb) and *kcc-2* 3′UTR (1.3 kb) were amplified by PCR from genomic DNA and were sub-cloned into the pPD95.75 vector. For the construction of the genomic *eor-1::mcherry* fusion gene, the *eor-1* genomic fragment (6.2 kb) without the stop codon was fused to *mcherry*. For the rescue experiment, *eor-1a* cDNA, *swsn-1a* cDNA, and *mau-2a* cDNA were amplified by PCR and sub-cloned into the pPD49.26 vector. The *unc-86* and *hsp-16.2* promoters were inserted upstream of the multi-cloning site of each plasmid. The constructs used for the nuclear spot assay, *yfp::htz-1* and *cfp::LacI*, were gifts from S. Mango. *cfp::LacI* was substituted by *mTurquoise2*, amplified by PCR. The *unc-86* and *htz-1* promoters were inserted upstream of the multi-cloning site of the *yfp::htz-1* and *mTurquoise2::LacI* constructs, respectively.

Transgenic strains were generated by microinjecting test DNA at a concentration of 10−50 ng/μL, along with a 2−20 ng/μL co-injection of the marker *Pmyo-2::gfp*, *Pmyo-2::mCherry*, *Punc-122::gfp*, or *Punc-122::mCherry*, and 0−70 ng/μL carrier DNA as described by Mello and Fire^66^. For nuclear spot assays, to avoid the contamination of *LacO* sites into the extrachromosomal array expressing *mTurquoise2::LacI*, the *Punc-86::yfp::htz-1* PCR product, the *Phtz-1::mTurquoise2::LacI* PCR product, and the co-injection marker *Punc-122::gfp* PCR product were injected into wild-type worms. Transgenes harboring the *abts-1b* promoter and *LacO* repeats were generated by injecting the 1.1 kb *abts-1b* promoter sequence, pSV2-DHFR8.32, which contains 256 copies of the *LacO* sequence, as well as the co-injection marker *Pmyo-2::gfp*.

### Quantitative fluorescent microscopy measurements

Mid-late L4 worms, identified by vulval morphology, were transferred to new NGM plates with OP-50 and incubated for 16–20 h at 20 °C. Staged adult worms were put on agar pads and anesthetized with 5 mM sodium azide. Cell body images of HSNs were obtained using a Carl-Zeiss LSM5 confocal microscope at a fixed setting. The sum of fluorescent intensities within the cell body was quantified by LSM5 Pascal software.

All data points represent the means of more than 30 worms. Asterisks indicate statistically significant differences (**p < 0.01). Statistical significance was determined by the Mann-Whitney U test for the comparison between wild-type and mutants, and for the rescue experiments.

Fluorescent images of transgenic worms harboring genomic *eor-1::mCherry* or *Pabts-1b::gfp* were obtained using a Carl-Zeiss LSM5 confocal microscope or an Olympus FV1000 confocal microscope.

### Heat shock experiments

The insertion line expressing *Pkcc-2c::gfp::kcc-2* 3′UTR was used for heat shock rescuing experiments, since the bright and stable expression of *Pkcc-2c::gfp* in HSNs allowed sensitized evaluation of *eor-1*-rescuing effect. *eor-1(cs28)* mutants carrying *Is*[*Pkcc-2c::gfp::kcc-2* 3′UTR] and *Ex*[*Phsp16.2::eor-1a*] were put on a 6 cm NGM plate and allowed to lay eggs for 3 h at 20 °C. 0 h or 72 h after egg-laying, a 2h-heat shock at 33 °C was delivered. The worms were cultured again at 20 °C and subjected to quantitative analysis of GFP expression in HSNs 96 h after egg-laying.

In each set of experiment, the mean fluorescent intensity of *Pkcc-2c::gfp* in HSNs of wild-type animals was assigned as 1.0, and each mean fluorescent intensity in *eor-1* mutants or transgenic animals were shown as relative intensity by comparing to wild-type animal. All data points represent the means of more than 30 worms. Asterisks indicate statistically significant differences (p < 0.01). Statistical significance was determined by the Mann-Whitney U test for the comparison between wild-type and mutants, and for the rescue experiments.

For the analysis of EOR-1::mCherry degradation, wild-type animals carrying *kyIs262* [*Punc-86::myr gfp, Podr-1::rfp*] and *Ex*[*Phsp16.2::eor-1a::mCherry*] were put on a 6 cm NGM plate and allowed to lay eggs for 3 h at 20 °C. Soon after egg-laying, a 2h-heat shock at 33 °C was delivered. The worms were cultured again at 20 °C and subjected to quantitative analysis of EOR-1::mCherry fluorescence in HSNs at 24 h, 48 h, 72 h after egg-laying.

The mean fluorescent intensity of EOR-1::mCherry in HSNs of wild-type animals without *Ex*[*Phsp16.2::eor-1a::mCherry*] was assigned as a relative intensity of 1.0. All data points represent the means of more than 28 worms. Asterisks indicate statistically significant differences (p < 0.01). Statistical significance was determined by the Mann-Whitney U test for the comparison between worms carrying the transgene (+Ex) and worms not carrying the transgene (−Ex).

### Nuclear spot assays

Nuclear spot assays were performed as described^39^, with some modifications. In brief, images were obtained using an Olympus FV1000 confocal microscope. Developmental stages were discriminated by distal tip cell migration and body length. HSN cells were identified based on YFP::HTZ-1 expression driven by the *unc-86* promoter and its relative location against gonadal primordium and coelomocytes visualized by the co-injection marker *Punc-122::gfp*. All nuclei of HSNs with mTurquoise2 spots were analyzed for the co-localization of YFP and mTurquiose2 dots.

All data points were collected from more than 30 worms. P values were calculated using the Fisher’s exact test. Asterisks indicate statistically significant differences (*p < 0.05. **p < 0.01).

### Data availability

Strains are available upon request.

## Electronic supplementary material

Supplementary information
