# Supplementary material for: Regulation of chromatin states and gene expression during HSN neuronal maturation is mediated by EOR-1/PLZF, MAU-2/cohesin loader, and SWI/SNF complex
Source: Sci Rep. 2018 May 21;8:7942. doi: 10.1038/s41598-018-26149-2 (PMC5962631; doi:10.1038/s41598-018-26149-2)
Supplement: Supplementary file 1 — Supplementary information [file 41598_2018_26149_MOESM1_ESM.pdf]

Supplementary information

Regulation of chromatin states and gene expression during HSN neuronal maturation is mediated by EOR-1/PLZF, MAU-2/cohesin loader, and SWI/SNF complex

Yoichi Shinkai[1, *], Masahiro Kuramochi[1, 2, 3], Motomichi Doi[1, *]

1. Molecular Neurobiology Research Group and DAI-Lab, Biomedical Research Institute, National Institute of Advance Industrial Science and Technology (AIST), Central 6, 1-1-1, Higashi, Tsukuba, Ibaraki, 305-8566, Japan
2. Present address: Graduate School of Frontier Sciences, The University of Tokyo, 5-1-5 Kashiwanoha, Kashiwa City, Chiba, 277-8561, Japan
3. Present address: AIST-UTokyo Advanced Operando-Measurement Technology Open Innovation Laboratory (OPERANDO-OIL), National Institute of Advanced Industrial Science and Technology (AIST), Chiba 277-0871, Japan

*. Corresponding authors

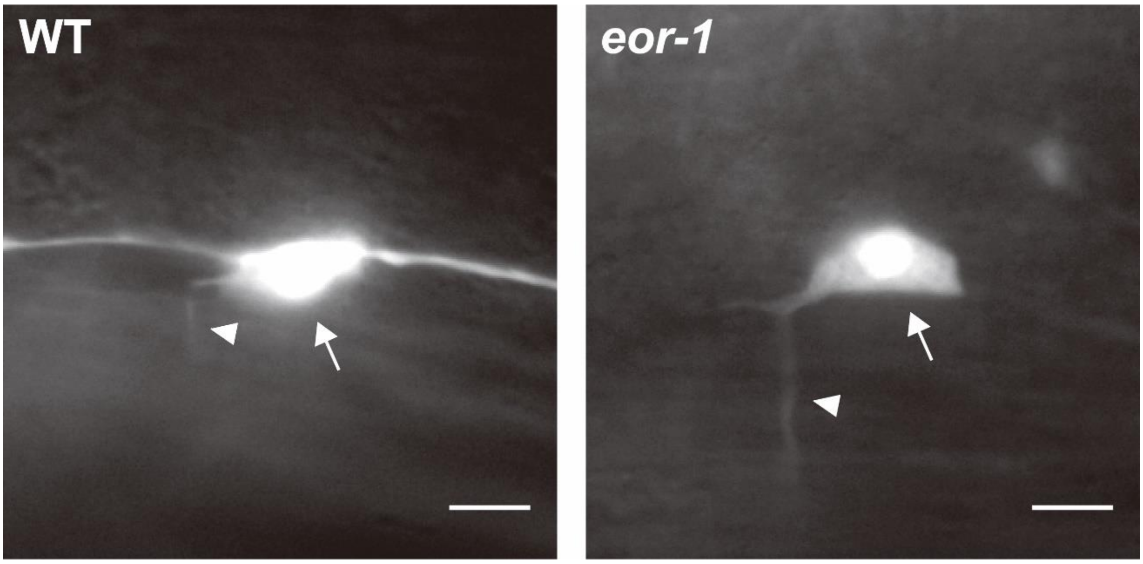

Supplementary Figure S1. Normal neurite elongation of HSNs in *eor-1* mutants.
Neurite elongation was examined in wild-type worms and *eor-1* mutants expressing *Pabts-1b::gfp*. Arrows indicate the cell body of HSN neuron. Arrowheads indicate a neurite from HSN neuron. In each image, the anterior side and the dorsal side of the worm are positioned to the left and the top. White scale bars indicate 5 µm.

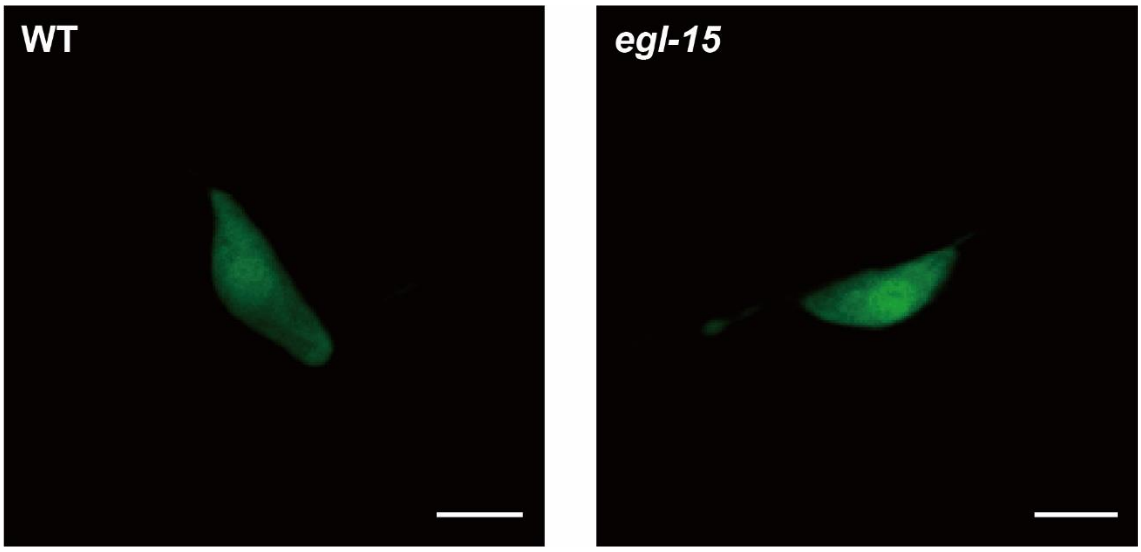

Supplementary Figure S2. The expression of *abts-1* gene in HSN neurons of the FGF signaling mutant.

Pabts-1b::gfp expression in HSNs was compared at the adult stage between wild-type worms and *egl-15(n484)* mutants. White scale bars indicate 5 μm.

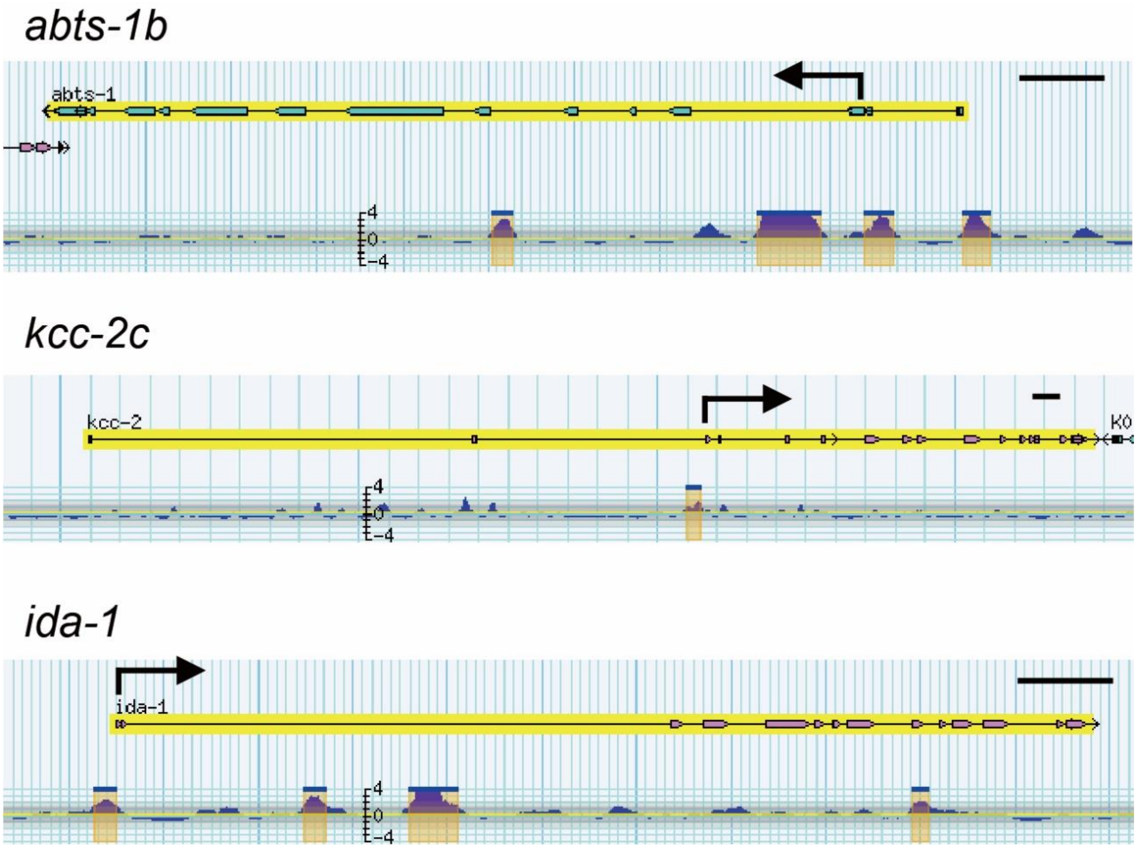

Supplementary Figure S3. ChIP sequencing analysis map of EOR-1 from modENCODE data on wormbase.org.

The regions highlighted by orange boxes indicate the significant enrichment of EOR-1 in the *C. elegans* genome. Arrows indicate transcription start sites in HSN neurons. EOR-1 was enriched on each promoter region of *abts-1*, *kcc-2*, and *ida-1*. Black scale bars indicate 1 kb.

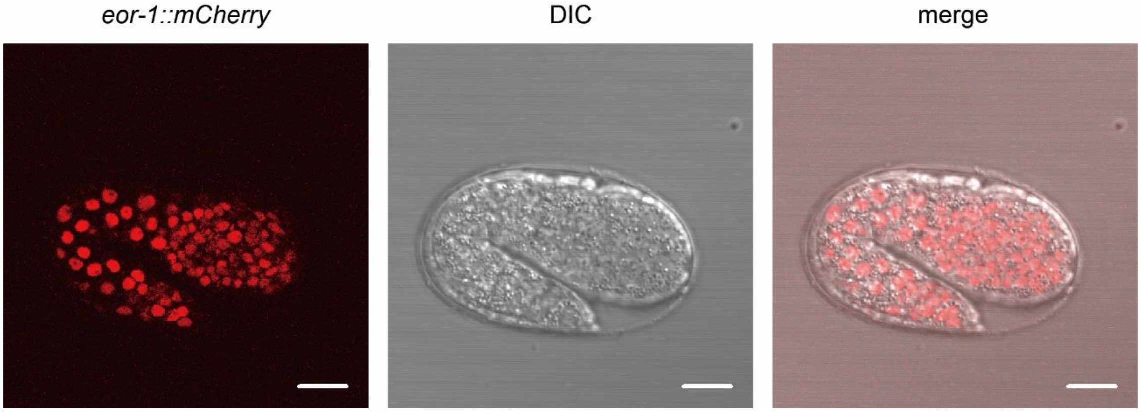

Supplementary Figure S4. The expression of *eor-1::mCherry* in embryo.

The pictures show the expression of *eor-1::mCherry* at the two-fold embryonic stage. White scale bars indicate 10 μm.

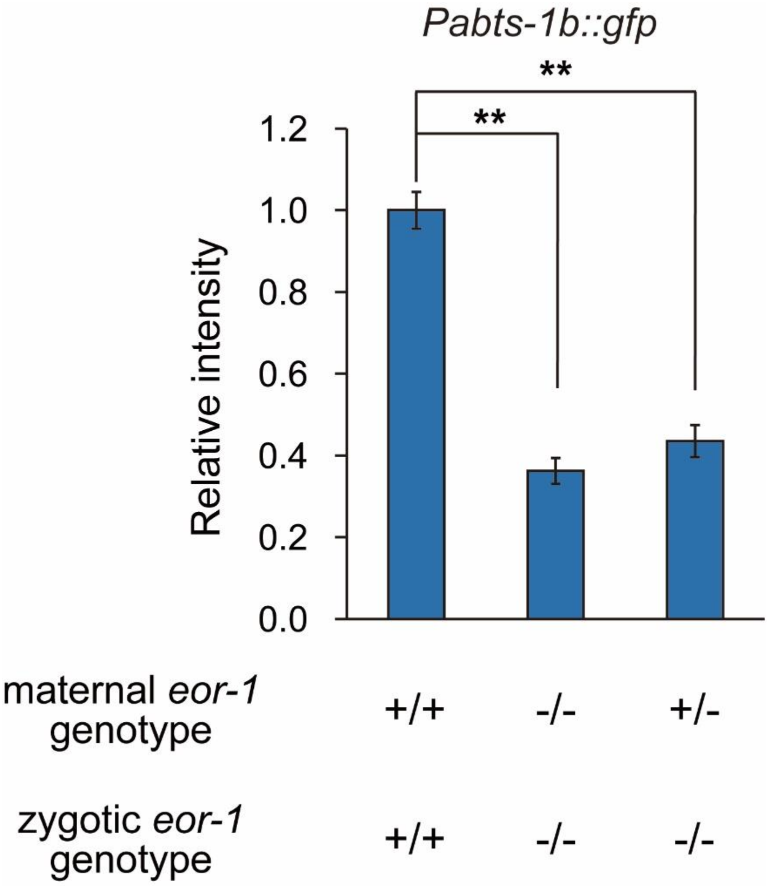

Supplementary Figure S5. Maternal effect of EOR-1 on the *abts-1* expression in HSNs. *Pabts-1b::gfp* expression in HSNs was compared at the adult stage among wild-type worms, homozygous *eor-1(cs28)* offspring from homozygous *eor-1(cs28)* mutants, and homozygous *eor-1(cs28)* offspring from heterozygous *eor-1(cs28)*/+ animals. Error bars indicate SEM (n ≥ 43, **p < 0.01).

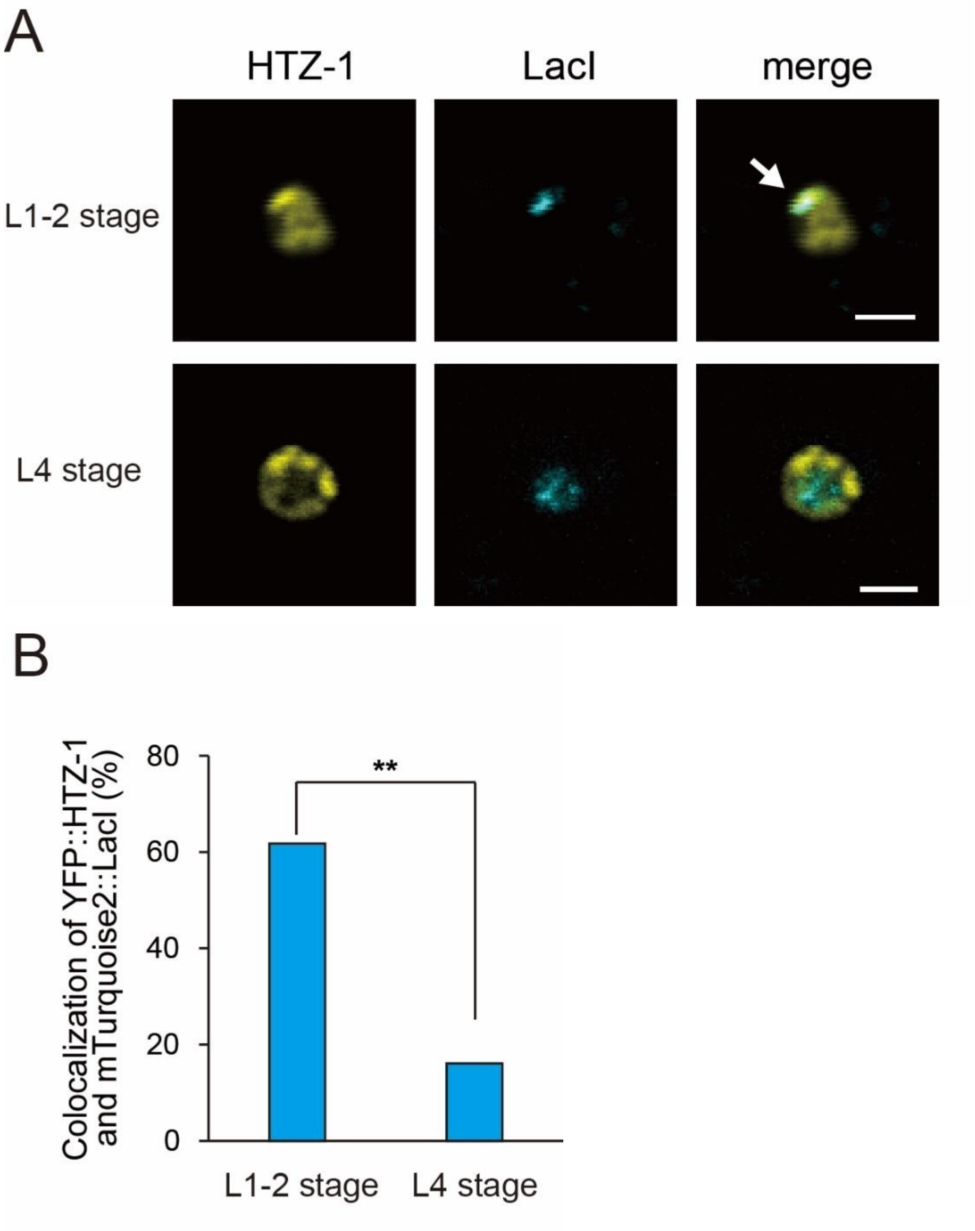

Supplementary Figure S6. Co-localization of HTZ-1 and the *abts-1b* promoter is attenuated as HSNs mature.

(A) Localization of YFP::HTZ-1 and extrachromosomal LacO reporter arrays carrying the *abts-1b* promoter in HSNs at the L1-2 or L4 larval stage. The extrachromosomal LacO reporter array was visualized by the binding of mTurquoise2::LacI. Arrow indicates co-localization of YFP::HTZ-1 and reporter arrays. White scale bars show 2μm.

(B) Quantitative data from the co-localization of YFP::HTZ-1 and reporter arrays carrying the *abts-1b* promoter in HSNs at the L1-2 or L4 larval stage. The percentage of the co-localization was analyzed. $n \geq 31$, $^{**}p < 0.01$.

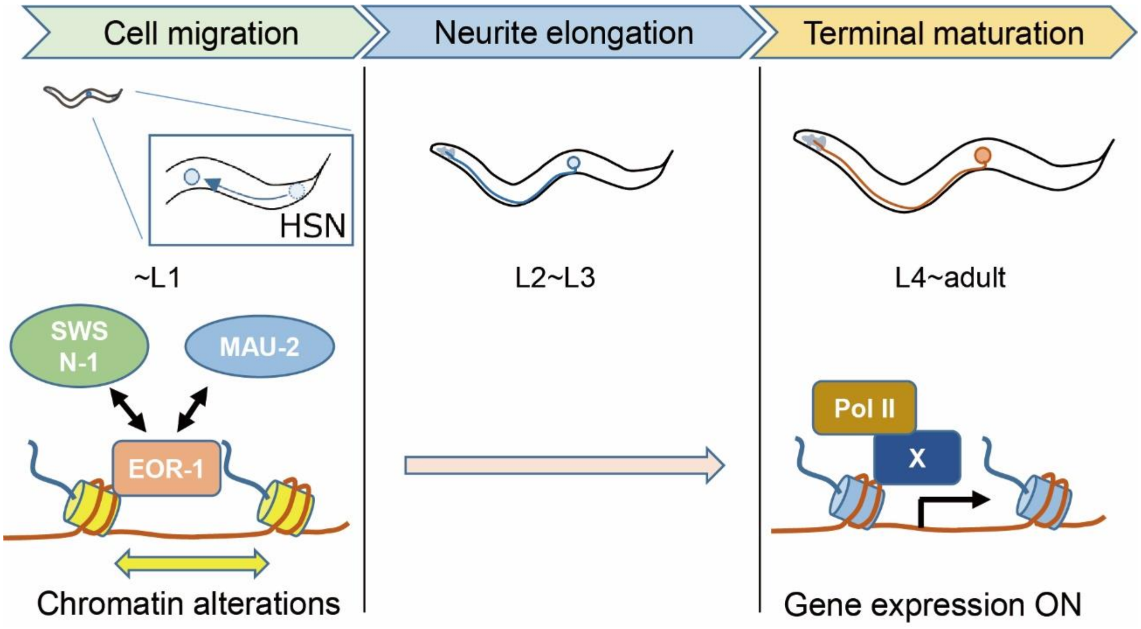

Supplementary Figure S7. A schematic illustration of the working model that EOR-1-dependent chromatin remodeling at the promoter region is a prerequisite for the later activation of gene expression.

The double-headed arrows indicate genetic interactions. EOR-1 directly binds to the promoter regions of genes required for HSN terminal maturation and modulates the chromatin state by the L1 larval stage. The pre-defined chromatin state would affect gene expression required for HSN terminal maturation, which would be initiated by an unidentified transcription factor or activator from the L4 larval stage onward.
